# The interplay between obesity, immunosenescence, and insulin resistance

**DOI:** 10.1186/s12979-024-00414-7

**Published:** 2024-02-05

**Authors:** Ghazaleh Shimi, Mohammad Hassan Sohouli, Arman Ghorbani, Azam Shakery, Hamid Zand

**Affiliations:** Department of Cellular and Molecular Nutrition, Faculty of Nutrition Science and Food Technology, National Nutrition and Food Technology Research Institute, Shahid Beheshti University of Medical Sciences, Tehran, 1981619573 Iran

**Keywords:** Obesity, Insulin resistance, Immunosenescence, Inflammation, Senescence-associated secretory phenotype, Senescence

## Abstract

Obesity, which is the accumulation of fat in adipose tissue, has adverse impacts on human health. Obesity-related metabolic dysregulation has similarities to the metabolic alterations observed in aging. It has been shown that the adipocytes of obese individuals undergo cellular aging, known as senescence. Senescence can be transmitted to other normal cells through a series of chemical factors referred to as the senescence-associated secretory phenotype (SASP). Most of these factors are pro-inflammatory compounds. The immune system removes these senescent T-cells, but immunosenescence, which is the senescence of immune cells, disrupts the clearance of senescent T-cells. Immunosenescence occurs as a result of aging or indirectly through transmission from senescent tissues. The significant occurrence of senescence in obesity is expected to cause immunosenescence and impairs the immune response to resolve inflammation. The sustained and chronic inflammation disrupts insulin's metabolic actions in metabolic tissues. Therefore, this review focuses on the role of senescent adipocyte cells in obesity-associated immunosenescence and subsequent metabolic dysregulation. Moreover, the article suggests novel therapeutic approaches to improve metabolic syndrome by targeting senescent T-cells or using senotherapeutics.

**Graphical Abstract:**

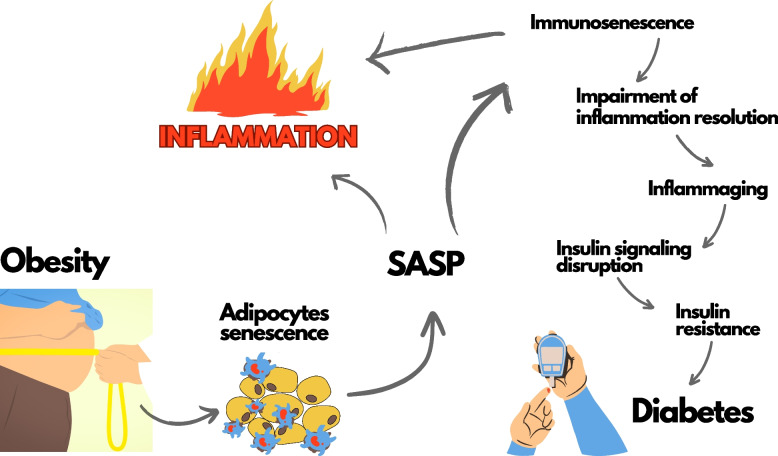

## Introduction

Obesity is a nutritional disorder characterized by an imbalance in energy intake and expenditure, and it is considered one of the most significant global health issues [1]. The origin of this disease is complex and involves multiple factors, including genetic, environmental, lifestyle, socioeconomic, cultural, and psychological aspects [2, 3].

The presence of obesity leads to an energy imbalance, characterized by an increase in both the size (hypertrophy) and number (hyperplasia) of adipocytes [4]. These changes are associated with abnormalities in the functioning of adipocytes, specifically in the endoplasmic reticulum (ER) and mitochondrial oxidative stress. These reactions lead to the generation of free fatty acids, inflammatory agents, and adipokines [5]. There is substantial evidence indicating that obesity is connected to a persistent low-level inflammation [6]. This inflammation is associated with various health issues including insulin resistance, diabetes mellitus, dyslipidemia, endothelial dysfunction, atherosclerosis, hypertension, cardiovascular diseases, cardiometabolic/metabolic syndrome, and cancer [4, 7].

Various pieces of evidence show that obesity is related to some conditions such as DNA damage, telomere attrition, metabolic dysregulation, and organelles stress. These changes can occur in several types of cells [8]. These cellular stresses can lead to cellular senescence, which has been identified as an important factor in age-related functional deterioration and the persistent low-grade inflammation that occurs during the aging process. Senescent T-cells release a series of soluble compounds including growth factors, extracellular proteases and pro-inflammatory factors known as the senescence-associated secretory phenotype (SASP) [9]. Senescent T-cells gradually accumulate in organisms as they age and cause chronic inflammation in adjacent tissues through the secretion of SASP. This process leads to a deterioration in organ function and contributes to the development of several age-related chronic conditions, including cardiovascular disease, endocrine and metabolic disorders, cancer, Alzheimer's disease, and autoimmune diseases [9–12]. During the process of chronological aging, various types of cells undergo cellular senescence, leading to the development of a senescent phenotype. One of these cells is the body's immune system cells, which undergo age-related changes and can contribute to the onset and progression of several age-related diseases [13, 14].

The immune system plays a crucial role in maintaining physiological balance, often referred to as homeostasis. As individuals age, their immune system undergoes certain changes, such as a gradual decrease in the ability to mount an effective immune response against foreign infections (which results in reduced vaccine effectiveness) and a tendency to generate excessive inflammatory reactions [15, 16]. The phenomenon is known as immunosenescence and is believed to be strongly linked to the development of obesity-related complications, such as insulin resistance and inflammation [14, 16, 17]. This is due to the secretion of the SASP factors by senescent adipocytes and immune cells, which leads to gradual changes in organ structure. Most types of cells in both the innate and adaptive immune systems undergo age-related alterations, particularly T-cells, which have a greater potential capacity for proliferatio [18]. Furthermore, chronic low-grade inflammation caused by senescence in adipose tissue and immune cells contributes to the development of obesity-related diseases [12, 19], involves a complex interplay of immune system responses, including acquired immunity mediated by T and B cells, as well as innate immunity involving macrophages [20, 21]. Previous studies have shown that obesity accelerated immunosenescence, independent of chronological aging [22, 23]. A study on mice fed a high-fat diet (HFD) also demonstrated the accumulation and increase of senescence-associated immune cells in visceral adipose tissue (VAT) [23]. These cells caused the production of large amounts of pro-inflammatory cytokines, eventually leading to chronic inflammation in the VAT. This chronic inflammation then resulted in insulin resistance [24, 25].

In essence, obesity and subsequent insulin resistance may independently cause immunosenescence and accelerate the progression of most chronic diseases through the acquisition of SASP by senescent immune cells. Thus, this review aims to investigate the relationship between obesity and insulin resistance with immunosenescence.

### The connection between obesity, insulin resistance, and the role of inflammation

Adipose tissue, as the primary energy reservoir of the body, stores energy in the form of triglycerides and releases energy in the form of free fatty acids (FFAs) and glycerol [26]. Adipose tissue has a significant impact on metabolic balance, not only by directly managing vast energy reserves, but also by releasing several bioactive proteins known as adipokines [27, 28]. Obesity has been shown to affect the synthesis of FFAs and adipokines, and it is associated with increased release of FFAs and abnormal secretion of adipokines [29, 30]. Both of these alterations associated with obesity can detrimentally affect insulin function, thereby establishing a probable link between obesity and insulin resistance.

One of the first theories for obesity-related insulin resistance was proposed by Randle et al., highlighteing the role of free fatty acids in disruption of insulin associated glucose metabolism [31]. Visceral and subcutaneous fat exhibit some variation in their contributions to this enlargement.Visceral fat exhibits reduced sensitivity to the antilipolytic impact of insulin, making it more prone to lipolysis compared to subcutaneous fat [32]. Moreover, FFAs that are released from visceral fat are transported through the portal circulation and promptly delivered to the liver. On the other hand, FFAs originating from subcutaneous fat are released into the systemic circulation [33]. The increased flow of FFAs from visceral fat, which then pass through the liver, may stimulate the production of new glucose (gluconeogenesis) and lead to insulin resistance in the liver [32–34].

It has been shown that the kinase cascade of insulin receptors, which begins with tyrosine phosphorylation, is disrupted by serine/threonine kinases activated by inflammation, including PKC, AKT, IKK, mTOR, S6K1, ERK1/2, and ROCK1. The phosphorylation of insulin receptor substrates (IRSs) and other downstream mediators of the insulin receptor by these kinases causes inflammation-induced insulin resistance in aging and metabolic diseases (see detailed review in [35]).

As noted earlier, the secretion of adipokines may serve as a link between obesity and insulin resistance. Adipose tissue is capable of modifying insulin action through the secretion of pro-inflammatory cytokines and other factors [36, 37]. The most extensively researched among these factors are adiponectin, leptin, plasminogen activator inhibitor-1 (PAI-1), tumor necrosis factor alpha (TNFα), and interleukin 6 (IL-6). Leptin, PAI-1, TNFα, and IL-6 levels are elevated in obese individuals and are associated with insulin resistance [37, 38]. There is evidence that TNFα and IL-6 may promote insulin resistance through pathways that hinder the translocation of glucose transporter 4 (GLUT4) to the plasma membrane [39]. Adiponectin, on the other hand, has insulin-sensitizing properties, and its levels have been found to be reduced in obese individuals [32, 37, 38]. Adiponectin may act as an insulin sensitizer primarily by stimulating fatty acid oxidation [30, 32, 38]. Production of these proteins appears to differ between subcutaneous and visceral adipose tissue depots. For example, the expression and secretion of IL-6 and PAI-1 are relatively greater in VAT, whereas the expression and secretion of leptin and adiponectin are relatively greater in subcutaneous fat [36].

Chronic inflammation in obesity without infection or autoimmunity is puzzling. The NOD-like receptor (NLR) family of innate immune cell sensors, such as the NLRP3 inflammasome, play a role in identifying non-microbial danger signals, which then activate caspase-1 and lead to the secretion of IL-1β and IL-18 (10.1038/nm.2279). IL-1β and IL-18 are pro-inflammatory cytokines released by immune cells infiltrating the adipose tissue of obese subjects. Human and animal studies have shown that obesity and insulin resistance are linked to an increase in the NLRP3 inflammasome in adipose tissue [40]. Therefore, it is indicated that there is a strong connection between NLRP3 inflammasome and obesity/insulin resistance.

### Immunosenescence

The immune system plays a pivotal role in the lifespan and overall health of humans. In older individuals, vaccines may not effectively stimulate the immune system. As individuals age, there is a growing decline in the effectiveness of both the body's natural immune responses. This leads to weakened defenses against pathogens and a higher risk of morbidity and mortality [41].

Biological cellular aging, or senescence, is a process that occurs in cells as a result of various cellular stresses. If cells with this condition have the ability to reproduce, they are permanently removed from the cell cycle. Despite having a stable metabolism, they no longer divide. During this phase, these cells enter a secretory phase in which they release a variety of chemical factors, such as cytokines, chemokines, and growth factors. The specific types of factors released can vary depending on the type of cell. However, inflammatory factors and growth factors are relatively common in various tissues [42].

The composition of SASP varies and depends on cell context. However, the pro-inflammatory components of SASP stimulate and instruct the immune and neighboring cells to eliminate senescent T-cells. Due to the pro-inflammatory nature of most of these factors, if cells undergoing biological aging are not properly eliminated, a mild to moderate systemic inflammation occurs in the organism. This inflammation is commonly seen in pathological conditions such as metabolic diseases or chronological aging process of the organism. One of the intriguing aspects of cellular aging is the transmission of this cellular condition to other tissues through SASP factors [43].

These released factors have the ability to affect the actions of nearby cells and tissues, as well as distant locations, through a process called paracrine senescence. The SASP can be transmitted to healthy cells in a manner that does not require direct cell-to-cell contact, inducing neighboring cells to undergo senescence. This occurrence is linked to the development of a secretory program that enhances cellular senescence and affects the surrounding tissue environment. The SASP can produce both positive and harmful outcomes, depending on the biological circumstances, and its individual elements are still being studied [44].

Immunosenescence refers to the cellular aging of immune cells. Like all tissues, this process can occur due to telomere erosion, DNA damage, activation of oncogenes, oxidative damage, and chronological aging of the organism [43]. Since senescent T-cells in other tissues are eliminated by the immune system's appropriate response, immunosenescence is more significant than cellular aging in other tissues [41]. As a result, when cellular senescence accumulates in immune cells, it increases senescence in the tissues.

Immunosenescence can be considered from two aspects. First, immune cells, like other tissues, can undergo senescence during the process of chronological aging [45]. Moreover, the accumulation of senescent T-cells, such as those caused by inflammation or metabolic disorders, can spread to immune cells through SASP in a paracrine manner.

The most studied aspect of aging in immune cells focuses on T-cells, primarily due to their long lifespan and their ability to proliferate within the immune cell populations. Telomere erosion is a hallmark of senescent T-cells. Research has demonstrated that human CD4 T-cells experience a loss of approximately 3,000 base pairs of telomeric sequences between the ages of 20 and 60 [46]. However, human T-cells do not reach a critical limit shorter than 5–6 kb, which may be attributed to telomerase activity in normal T-cells. It is interesting that telomerase activity is decreased in patients with rheumatoid arthritis [47]. Opposite to telomere-associated senescence, premature senescence is triggered in cells, including immune cells, by cellular stressors such as oxidative stress, mitochondrial dysfunction, epigenetic alterations, perturbed proteostasis, persistent DNA damage, and oncogenes. This type of senescence is more involved in immunosenescence compared to telomere-associated senescence [43].

Decline in the DNA repair system is also one of the culprits of aging immune cells. One study revealed that a mutation in the DNA excision-repair gene, Ercc1, causes the accumulation of DNA damage lesions and a decrease in the proliferation capacity of T regulatory cells, helper T-cells, and cytotoxic T-cells [48]. There is a shift in the metabolic pathways of aging T-cells from glycolytic metabolism towards pentose phosphate and NADPH production. This change in the metabolic process prevents older T-cells from receiving signals related to oxidation [49]. Activation of 6-phosphofructo-2-kinase/fructose-2, 6-biphosphatase 3 (PFKFB3) also decreases in aged T-cells, resulting in reduced glucose utilization through glycolysis [50].

Age also impacts the differentiation and number of B cells. The B cells of aged individuals have a reduced level of the helix-loop-helix protein, transcription factor 3/E47. This transcription factor is involved in B and T-cell development. The decrease in E47 levels in older individuals may be due to the instability of its mRNA caused by the expression of the inflammatory miRNAs 16 and 155 [51, 52].

Innate immune cells also undergo changes with aging. Tool-like receptors expression pattern are alters in dendritic cells of aged subjects as antigen presenting cells (APCs). Healthy older adults have more cytotoxic natural killer cells than their frail counterparts. The phagocytic capacity of neutrophils, as well as their respiratory burst, declines with aging. The production of cytokines, chemotaxis, and phagocytosis decreases in macrophages during aging [53].

These results show that senescence induced by metabolic and inflammatory diseases may lead to immunosenescence in immune cells. This immunosenescence is responsible for accelerating inflammaging and other age-associated disorders. Therefore, age-induced senescence in immune cells or transmission from senescence in other non-immune cells may be the primary factors contributing to biological aging.

### Immunosenescence contributes to the disruption of resolving inflammation

Immunosenescence leads to dysregulation of the immune response, resulting in chronic inflammation. It modifies both the innate and adaptive immune responses, resulting in an expansion of memory T-cells, a diminished capacity to react to antigens, and a sustained low-grade inflammation [45]. Inflammation resolution is an active and coordinated process that occurs in response to inflammation, aiming to limit tissue damage and promote repair. When the resolution program fails, inflammation persists, leading to chronic inflammatory diseases such as cardiometabolic syndrome [54].

The resolution of inflammation involves the initiation of pro-resolving mediator production, the clearance of dead cells, and functional changes in immune cells. During the initiation phase, pro-inflammatory cytokines and mediators are involved in the steps of inflammation, such as inflammatory eicosanoids, while simultaneously promoting pro-resolving pathways. Then, pro-resolving mediators are produced to counteract the pro-inflammatory signals and promote the resolution of inflammation. The resolution of inflammation involves several steps, including the production of specialized pro-resolving lipid mediators (SPMs) and the activation of pro-resolving pathways. The biosynthesis of SPMs, such as lipoxins or resolvins, typically requires the involvement of arachidonic acid 5-lipoxygenase and various types of arachidonic acid 12- and 15-lipoxygenating paralogues. These SPMs are believed to be formed through successive oxidation of polyenoic fatty acids, such as arachidonic acid, eicosapentaenoic acid, or docosahexaenoic acid [55].

The next step in resolving inflammation is the clearance of dead cells, which occurs during the inflammatory phase. For example, during a process known as efferocytosis, macrophages engulf neutrophils that have infiltrated inflamed tissues. These macrophages initiate the release of mediators to resolve inflammation and potentiate resolution [56]. In the final stage, functional changes occur in immune cells to promote the resolution of inflammation. These steps are crucial for the active and highly coordinated process of resolving inflammation, which aims to limit tissue damage and promote repair. In cases of obesity, there is a shift in the polarization of M1 macrophages, resulting in persistent inflammation and insulin resistance. This polarization shift occurs due to factors such as TLR-4, hypoxia, free fatty acids, and pro-inflammatory cytokines. M1 macrophages are associated with insulin resistance, while anti-inflammatory polarization, M2, is linked to insulin sensitivity. Research has demonstrated that peroxisome proliferator-activated receptor gamma (PPARγ) contributes to the transition of M1 to M2 macrophages. Natural ligands of PPARγ, such as omega-3 fatty acids, may have a beneficial effect in improving metabolic dysregulation [57, 58].

### The connection between obesity and immunosenescence

Metainflammation is a persistent, mild inflammation present in the body that is linked to several metabolic conditions including obesity, type 2 diabetes, cardiovascular disease, and non-alcoholic fatty liver disease. It is identified by an unequal balance between pro-inflammatory and anti-inflammatory pathways, resulting in ongoing inflammation. Metainflammation is initiated by a range of factors including excessive food intake, obesity, lack of physical activity, and exposure to environmental pollutants. These factors cause the body to produce pro-inflammatory cytokines like TNFα, IL-6, and C-reactive protein (CRP). The effects of metainflammation are extensive and can impact various organ systems. In fat tissue, metainflammation results in insulin resistance and the release of adipokines that contribute to inflammation [59].

Obesity has a similar impact on cellular and molecular processes as aging [60]. Since obese individuals tend to be older in their biological age, the phrase “adipaging” has been employed to define the inflammation linked to chronic obesity [61]. Adipose tissue aging accelerates due to obesity, and dysfunctional adipose tissue in obesity may result in telomere attrition and higher oxidative stress, which could negatively affect mitochondria and genomic stability [62]. During adipaging, increased production of reactive oxygen species may cause a reduction in immune system function. Due to this, the homeostatic networks associated with inflammation become dysregulated, leading to alterations in IL-6, TNFα, and adipokine secretion [63].

Chronic low-grade inflammation in obesity is associated with senescent T-cells that exhibit SASP and secrete pro-inflammatory cytokines without any antigenic stimulation. The accumulation of senescent T-cells can occur as a result of a dysfunctional immune system or immunosenescence caused by chronic inflammation [64]. Thus, obesity may induce immunosenescence [42] and cause a reduction in innate immunity including decreased neutrophil and macrophage activation, as well as cytotoxic activity of natural killer (NK) cells. It can also lead to changes in lymphocyte responses, such as an increase in anergic memory T-cells, a decline in naïve T-cells, and exhaustion of helper or cytotoxic T-cells and B lymphocytes (see detailed review in [65]).

In obese individuals, VATs have elevated levels of major histocompatibility complex class II (MHCII) and costimulatory molecules. As obesity progresses, antigens (likely self-peptides) on MHCII molecules are known to stimulate T-cell proliferation and differentiation into specific subclasses of inflammatory effectors. The persistence of VAT inflammation can be due to this factor [66]. Moreover, obese individuals have elevated levels of leptin, which can trigger the activation of T-cells and facilitate their transformation into the interferon‐gamma (IFN‐γ) -producing type 1 T helper (Th1) phenotype [42]. There is also evidence that circulating leptin levels and leukocyte telomere length are inversely associated, demonstrating the complex relationship between leptin and markers of cellular aging [67]. In contrast, adiponectin, which decreases in obesity, has the potential to reduce telomere attrition by suppressing inflammatory signaling and insulin resistance [67].

Obesity can also cause a reduction in thymic function by converting thymic fibroblasts to adipocytes due to lipid accumulation. This can be a significant indicator of immunosenescence [64]. Accumulation of lipids leads to an increase in IL-6, leukemia inhibitory factor, and oncostatin M, which impede thymic functions and trigger thymocyte apoptosis [66]. When obesity decreases thymic output, peripheral T-cells undergo extensive homeostatic proliferation, which may result in T- cell senescence. Consequently, the number of naïve T-cells decreases, leading to an increased reliance on memory T-cells. The loss of naïve T-cells results in diminished cell-mediated immunity and a related increase in susceptibility to infectious agents. In addition, obesity has the potential to limit the diversity of T-cell receptors (TCRs) [42].

Senescent T-cells exhibit telomere shortenings, phenotypical change (lower CD28 expression), and cell cycle arrest. Yet, exhausted T-cells refers to effector T-cells with reduced cytokine expression and function, and they are resistant to reactivation. One of the signs of T-cell senescence is the presence of programmed death-1 (PD-1). The cell surface molecule PD-1, mediates immune suppression. Individuals with obesity have a higher level of PD-1 expression on their T-cells [66]. The B cells and macrophages in VAT can also strongly express programmed death-ligand 1 [23]. Chronic inflammation in obesity may induce autoreactive or hyperactive T-cell responses, which could potentially be prevented by PD-1 [68]. In this regard, studies have shown that obesity is associated with the effectiveness of PD-1/ PD-L1 inhibitors in patients and mice with cancer. Additionally, PD-1 blockade has been found to be effective against senescent T-cells [68, 69].

CD153 + PD-1hi CD4 T-cells are senescence-associated T-cells that were initially identified as a distinct subset of T-cells. They emerge during aging or obesity and are a part of the process of chronic local inflammation [66]. B-cells are partly responsible for the generation of CD153 + PD-1hi CD4 T-cells in VAT, which in turn contribute to inflammation and metabolic disorders in obesity. [23]. CD153 + PD-1hi CD4 T-cells can produce a significant quantity of osteopontin (OPN). OPN is a potent chemoattractant for macrophages that accelerates macrophage migration, promotes M1 macrophage polarization, activates effector T-cells, and inhibits the function of regulatory T-cells. Activating B-cells to produce immunoglobulins (Igs), suppressing IL-10 production by B cells, and dysfunction of regulatory B cells (Bregs) are other effects of OPN. All of the mentioned functions of OPN can contribute to the perpetuation of chronic inflammation [23, 66]. Therefore, it was proposed that senescent T-cells could be a promising treatment for obesity-related immunometabolic disorders. Based on a study, a vaccine targeting CD153 was shown to reduce senescent T-cells in the visceral fat of obese mice and improve insulin resistance [70].

There appears to be a complex phenotypic change in T-cells after weight reduction in individuals with obesity. CD153 + PD-1hi CD4 T-cells have been shown to have a long lifespan and to be resistant to elimination after weight loss [61]. According to research, weight reduction in obese mice resulted in the accumulation of CD4 T-cells in the VAT. These cells, in turn, recruited and maintained pro-inflammatory macrophages in the VAT, even after the body weight was normalized [71]. In addition, in another study, mice with a history of obesity regained weight very quickly in a weight gain–loss-regain model. The study concluded that immune cells can retain a memory of obesity and store it in CD4 + T-cells, which ultimately results in inevitable weight regain [72]. Thus, it is postulated that senescent CD4 T-cells could potentially contribute to the negative effects of obesity.

Both obesity and aging cause similar alterations in the activity of transcription factors, such as transcription factor 3/E47, which are involved in class switching. These changes can be partially reversed through weight loss. Due to a redistribution of B cell subsets in the circulating B cell pool towards inflammatory B cells called double negative B cells, vaccine-specific antibodies are reduced in obese and elderly individuals. Interestingly, obese subjects exhibit an increase in senescence markers and expression of SASP components in B cells, which decrease with weight loss [73].

Another factor to consider is that obesity can alter the physiology of NK cells, which in turn play a role in the dysfunction of adipose tissue related to obesity [74]. NK cells enhance the secretion of pro-inflammatory cytokines by drawing macrophages to the adipose tissue. Afterwards, the infiltration of macrophages may result in the occurrence of chronic inflammation and the development of insulin resistance [75]. A significant amount of research has been conducted on the topic of obesity prevention, including studies on body weight reduction through caloric restriction or bariatric surgery, as well as the impact of physical activity on NK cells. While most studies have shown a positive effect of caloric restriction (CR) on NK cell function [76], a few have shown negative results. These negative results include decreased killing activity, impaired maturation, and reduced IFN-γ production of NK cells in non-obese mice or obese women [77, 78]. In addition to the effects of CR on the functionality of NK cells, a study conducted by Moulin CM et al. (2011) showed that six months after bariatric surgery, the cytotoxicity and cytokine production of NK cells increased significantly in obese patients [79]. However, in a different study, it was found that 12 months after gastric bypass surgery, obese patients showed a decrease in the expression of the activation marker CD69 and the Fas antigen CD95 [80]. Different surgical methods and the degree of weight loss may contribute to these conflicting findings. Finally, Barra NG and colleagues (2017) demonstrated the impact of physical activity on NK cells. Their study revealed that engaging in higher-intensity interval exercise led to an increase in the number and activity of blood NK cells. Additionally, this exercise regimen resulted in a reduction in the burden of lung cancer in obese mice [81]. According to this finding, activating NK cells during exercise may reduce complications related to obesity (reviewed in [75]). Totally, it appears that fat loss from diet and exercise can reduce inflammation in adipose tissue and improve immune function and insulin sensitivity.

The first immune cells to enter the adipose tissue of obese individuals are neutrophils. Activation of neutrophils leads to the release of multiple inflammatory factors that attract other immune cells, resulting in additional inflammation in obesity. Circulating neutrophils are higher in obese individuals. In general, neutrophils in obese individuals appear to exhibit an activated phenotype, as evidenced by their high production of reactive oxygen species (ROS) (see the detailed review in [82]). Other functions of neutrophils, such as phagocytosis, chemotaxis, and the formation of neutrophil extracellular traps (NETs), are still unknown. It is surprising that activated neutrophils fail to control infections in obese individuals [82]. One possible explanation for the lack of effective antimicrobial activity in neutrophils of obese individuals is that these neutrophils can trigger paracrine senescence in neighboring cells by means of ROS-mediated telomere dysfunction [83].

Moreover, obesity-induced inflammation activates immune cells such as macrophages. Some mechanism proposed for this phenotypic changes in immune cells. Obesity may cause epigenetic alterations, and change expression of genes that respond to inflammation without changing the DNA sequence itself. For example, obesity can alter DNA methylation or histone modification of genes that are responsible for B cell development or T-cell differentiation in thymus, and also affects microphage polarization. These abnormalities in the epigenome lead to changes in the basic mechanisms of immune function, which are implicated in the onset and progression of common metabolic disorders [84].

Together, it is suggested that obese individuals, compared to those who are not obese and have the same chronological age, are more likely to experience premature signs of aging. Therefore, obesity is a potential indicator of immunosenescence, which leads to alterations in the immune profile.

### The connection between insulin resistance and immunosenescence

Proper immunological and inflammatory responses are essential for survival. The antagonistic pleiotropy theory of aging proposes that while the immune system's acute responsiveness to challenges is necessary for tissue repair and infection resistance, the chronic inflammatory responses seen in the aging immune system contribute to the polarization of cell-mediated immune responses and metabolic deregulation in older individuals. This immunological dysfunction is characterized by insulin resistance and plays a role in the development of degenerative diseases commonly associated with aging, such as type 2 diabetes, cardiovascular disorders, and neurological disorders [85].

Ageing leads to the deregulation and decline of several components of the immune system. The decline in immunity leads to a higher occurrence and severity of infectious diseases, cancer, and autoimmune disorders. This also contributes to a higher incidence of illness and mortality among the elderly population [86, 87]. An essential consequence of the impaired immune response in aging is the emergence of chronic low-level inflammation, which can serve as the underlying factor for various metabolic disorders, such as insulin resistance. This occurs due to the dysregulation and overproduction of pro-inflammatory cytokines, leading to inflammaging [88]. Various types of immune cells are responsible for secreting cytokines during the inflammatory response. Th1 cells produce IL-2 and IFN-γ and play a role in cell-mediated immune responses. On the other hand, Th2 cells are responsible for Th2 immunity by producing cytokines such as IL-4 and IL-5, which stimulate antibody responses. Monocytes and macrophages are capable of producing both Th1 and Th2 cytokines. Evidence from research conducted on mice and humans indicates that a transition from Th1 to Th2 immunity during the aging process plays a significant role in immunosenescence [89–91]. The decline in Th1 function that occurs with age is believed to be responsible for the shift towards Th2 immunity and may partially contribute to the higher occurrence of diseases associated with inflammation related to aging, such as insulin resistance [14]. Macrophages also play a significant role in creating this change. Stimulated macrophages from older donors exhibit a significant increase in Th1 and Th2 cytokines, including the pro-inflammatory molecules TNFα and IL-6, as compared to those from young donors [92]. Elevated concentrations of IL-6 can be observed in the blood of older individuals, even in the absence of external stimulation or inflammation. This suggests the presence of detectable chronic inflammation in the elderly [93]. The elevation of pro-inflammatory mediators, such as IL-6 and TNFα, is believed to contribute to the onset of various age-related conditions, including diabetes mellitus and insulin resistance [94]. Elevated inflammatory state directly correlates with a range of negative aging consequences. Chronic activation of innate immunity is a characteristic feature of the insulin resistance syndrome, resulting in low-level inflammation that is both caused by and contributes to immunosenescence. Indeed, the levels of inflammation can be served as a predictor for the progression of diabetes and future morbidity [95]. The aging process is associated with higher levels of the suppressor of cytokine signaling (SOCS) family, which is responsible for reducing inflammation [96]. These proteins have the ability to directly hinder insulin pathways, thus contributing to insulin resistance and obesity. Obesity is linked to insulin resistance and can lead to the development of diseases such as type 2 diabetes. The etiology and pathogenesis of insulin resistance related to obesity are believed to be significantly influenced by systemic inflammation. Population studies have shown a link between the production of pro-inflammatory cytokines and metabolic dysregulation. Elevated levels of TNFα, IL-6, and CRP are observed in individuals who are both insulin resistant and obese. The excessive production of these cytokines in individuals with insulin resistance syndrome is linked to an increased risk of developing type 2 diabetes mellitus and cardiovascular diseases [95, 97]. Furthermore, it is postulated that the presence of insulin resistance, along with concurrent hyperinsulinemia, hyperglycemia, and increased production of inflammatory cytokines, may contribute to vascular inflammation and facilitate the progression of atherosclerotic cardiometabolic disease. Furthermore, Hotamisligal et al. indicated a link between obesity, heightened production of the pro-inflammatory cytokine TNFα, and diminished insulin sensitivity [98]. In addition, research has demonstrated that both type 2 diabetes and obesity impair the functions of B cells in both young and elderly individuals. B cells from both young and elderly individuals with obesity also promote the generation of the inflammatory cytokines IL-17 and IFN-γ in T-cells [99]. It has been proposed that if many diseases share a common root cause, such as cellular senescence, there is potential for the development of a singular pharmaceutical intervention capable of treating multiple diseases such as obesity-related insulin resistance, diabetes and cardiovascular diseases [100].

Insulin resistance has been linked to the presence of several pro-inflammatory cytokines, including IL-6, as well as SOCS, ER stress, and the inhibitor of nuclear factor kappa-B kinase subunit beta (IKKB) and c-Jun N-terminal kinase (JNK) signaling pathways [101, 102]. These findings establish a correlation between cytokines produced by adipose tissue, the immune system, and insulin resistance [59]. There is a hypothesis that insulin resistance could be accelerated or triggered by an innate immune response. This reaction involves an increase in Th1 and Th2 cytokines, including TNFα and IL-6, as well as pro-inflammatory adipose-derived cytokines such as leptin and resistin. These substances are found in the adipose tissue of obese individuals.

### Targeting senescent T-cells as a new therapeutic strategy in insulin resistance-associated diseases

Immunosenescence is accompanied by disruption of insulin signaling. Targeting senescent T-cells should play a crucial role in the new pharmacological strategy for treating cardiometabolic syndrome and its related complications. Senotherapeutics is an interesting area that focuses on targeting senescent T-cells, including senolytic, senomorphic, SASP inhibitors, and inducers of autophagy. Senolytics selectively kill senescent T-cells; however, senomorphics alter the phenotype and behavior of senescent T-cells. SASP inhibitors target the production of SASP and promote an anti-inflammatory state in the organism. Autophagy inducers stimulate cells to recycle their components and clear senescent T-cells through autophagy. These compounds potentially can be used to improve insulin resistance associated with age and obesity [103, 104].

Moreover, drugs that enhance the resolution of inflammation by the immune system have gained increasing attention in recent years as a potential therapeutic approach for various inflammatory diseases. SPMs such as resolvins, protectins, and maresins are generated from polyunsaturated fatty acids and play a key role in resolving inflammation by promoting clearance of inflammatory cells and debris, reducing pro-inflammatory cytokine production, and stimulating tissue repair. SPMs have shown efficacy in various animal models of inflammation including arthritis, asthma, and colitis [105]. Annexin A1 (ANXA1) mimetics, a protein involved in the resolution of inflammation, have been effective in animal models of inflammation including arthritis, colitis, and sepsis. Glucocorticoids enhance the resolution of inflammation by promoting the expression of SPMs and ANXA1 [106]. Aspirin inhibit the production of pro-inflammatory mediators, contributing to the resolution of inflammation [107].

## Conclusion

Obesity, characterized by the excessive accumulation of body fat, induces senescence in fat tissue, leading to adipocyte dysfunction. Furthermore, there is a direct relationship between obesity and the accumulation of senescent T-cells in other organs. The significant presence of senescent T-cells in obesity is expected to boost the production of SASP and exacerbate the inflammatory state, leading to immunosenescence, which in turn impairs the immune system's ability to effectively remove inflammation. As a result, the obesity-associated immunosenescence can disrupt metabolic processes and contribute to the development of cardiometabolic syndrome.

Finally, the importance of senescent T-cells in obesity-related immunosenescence and subsequent metabolic disorders can lead us to consider that targeting senescent T-cells for therapeutic purposes may be a useful pharmacological strategy to treat obesity-associated complications in the future.

## Data Availability

No datasets were generated or analysed during the current study.

## Abbreviations

SASP: Senescence-associated Secretory Phenotype
ER: Endoplasmic Reticulum
DNA: Deoxyribonucleic Acid
HFD: High Fat Diet
VAT: Visceral Adipose Tissue
FFAs: Free Fatty Acids
PKC: Protein Kinase C
IKK: IκB kinase
mTOR: Mammalian Target of Rapamycin
ERK 1/2: Extracellular Signal-regulated Kinase ½
S6K1: Ribosomal Protein S6 Kinase Beta 1
ROCK1: Rho-kinase 1
PAI-1: Plasminogenactivator Inhibitor-1
TNFα: Tumor Necrosis Factor Alpha
IL-6: Interleukin 6
GLUT4: Glucose Transporter 4
NLR: NOD-like receptor
NLRP3: NLR Family Pyrin Domain Containing 3
NADPH: Nicotinamide adenine dinucleotide phosphate
PFKFB3: 6-Phosphofructo-2-kinase/fructose-2,6-biphosphatase 3
APC: Antigen Presenting Cells
SPMs: Specialized Pro-resolving Lipid Mediators
TLR-4: Toll-like Receptor 4
PPARγ: Peroxisome Proliferator-activated Receptor Gamma
CRP: C-reactive Protein
NK cells: Natural Killer Cells
MHCII: Major Histocompatibility Complex Class II
IFN‐γ: Interferon‐gamma
Th1: Type 1 T Helper
TCR: T-cell Receptor
PD-1: Programmed Death-1
PD-L1: Programmed Death-Ligand 1
OPN: Osteopontin
Igs: Immunoglobulins
Bregs: Regulatory B cells
CR: Caloric Restriction
ROS: Reactive Oxygen Species
NETs: Neutrophil Extracellular Traps
SOCS: Suppressor of Cytokine Signaling
IKKB: Inhibitor of Nuclear Factor Kappa-B Kinase Subunit Beta
JNK: C-Jun N-terminal Kinase
ANXA1: Annexin A1
