# Correction: The interplay between obesity, immunosenescence, and insulin resistance

**DOI:** 10.1186/s12979-024-00438-z

**Published:** 2024-05-15

**Authors:** Ghazaleh Shimi, Mohammad Hassan Sohouli, Arman Ghorbani, Azam Shakery, Hamid Zand

**Affiliations:** Department of Cellular and Molecular Nutrition, Faculty of Nutrition Science and Food Technology, National Nutrition and Food Technology Research Institute, Shahid Beheshti University of Medical Sciences, Tehran, 1981619573 Iran

**Correction** **:** **Immun Ageing 21, 13 (2024)**

**https://doi.org/10.1186/s12979-024-00414-7**

Following publication of the original article [1], the authors reported a typo found in the graphical abstract, the word “inflamation “ needs to be corrected to “inflammation “. The corrected graphical abstract is presented below.


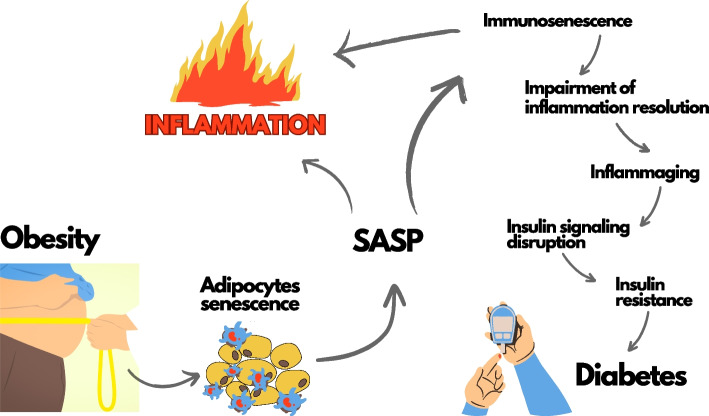


The original article [1] has been updated.
